# The softfoot pro at the cybathlon: kinematic, metabolic, and user performance evaluation

**DOI:** 10.1186/s12984-025-01862-y

**Published:** 2026-01-30

**Authors:** Simone Mora, Matteo Crotti, Anna Pace, Giorgio Grioli, Antonio Bicchi, Maura Casadio, Manuel Giuseppe Catalano

**Affiliations:** 1 Soft Robotics for Human Cooperation and Rehabilitation, Istituto Italiano di Tecnologia, Via S. Quirico 19D, Genova, 16163 Italy; 2 Department of Information Engineering and Research Center “Enrico Piaggio”, University of Pisa, Largo Lucio Lazzarino, 1, Pisa, 56122 Italy; 3 Department of Informatics, Bioengineering, Robotics and Systems Engineering, University of Genova, Via All’Opera Pia 13, Genova, 16145 Italy

**Keywords:** Foot prosthesis, Adaptive foot, Metabolic analysis, Kinematic analysis, Cybathlon

## Abstract

**Background:**

Prosthetic feet are essential for restoring independent mobility in individuals with lower-limb loss. However, most commercial prosthetic feet rely on elastic elements and rigid, flat soles, limiting adaptability to uneven terrain and compromising user stability. Originally developed for robotic applications, the SoftFoot introduced an adaptive sole architecture inspired by the biomechanics of the human plantar fascia to improve ground conformity and gait stability. Building on this concept, we introduced the SoftFoot Pro at Cybathlon 2024 — a prosthetic counterpart that integrates a compliant adaptive sole with energy storage capabilities at the ankle joint through an agonist–antagonist mechanism. This design emulates the synergistic action of muscles, tendons, and the plantar fascia in the human shank-ankle-foot complex. This study evaluates the kinematic and metabolic performance of the SoftFoot Pro.

**Method:**

During a dedicated pre-competition training session, the official SoftFoot Pro team pilot completed the Cybathlon Leg Race track twice using three prosthetic feet: (i) the Triton foot (energy storage only), (ii) the original SoftFoot (adaptive sole only), and (iii) the SoftFoot Pro. Kinematic and metabolic data were collected using the Xsens MVN Awinda and Cosmed K5 system. The evaluation was complemented by questionnaires assessing locomotor performance, usability, cognitive load, and user experience.

**Results:**

The SoftFoot Pro demonstrated greater ankle mobility than the original SoftFoot and the Triton across various tasks. Stride length and gait velocity were comparable to the Triton and higher than with the original SoftFoot. The SoftFoot Pro revealed the fastest circuit completion time, with a metabolic cost of transport comparable to the Triton and lower than the original SoftFoot. Questionnaires reported higher perceived mobility and lower cognitive and physical effort with the SoftFoot Pro, compared to both the original SoftFoot and the Triton, highlighting its functional and user experience advantages.

**Conclusions:**

This explorative, single-subject study quantitatively evaluated adaptive prosthetic feet in Cybathlon tasks simulating daily activities. Integrating an ankle joint with an agonist–antagonist energy recycling system improved mobility and reduced mental and physical effort, matching the performance of commercial carbon fiber feet while preserving the adaptive sole’s advantages. The Cybathlon was the catalyst for advancing the innovation and validation of our adaptive prosthesis.

**Supplementary Information:**

The online version contains supplementary material available at 10.1186/s12984-025-01862-y.

## Background

Living with a disability is a severe condition for many people, as they often do not have equal access to health care, education, and employment opportunities, and frequently experience exclusion from everyday life activities [1]. According to [1], approximately $18\%$ of the global population suffers from some form of disability, with limb loss being a prevalent condition that significantly affects an individual’s ability to perform daily tasks. This often leads to physical inactivity, resulting in cardiovascular diseases, muscle atrophy, joint stiffness, obesity, and depression [2]. Around 38 million people worldwide are affected by major amputations, with $85\%$ of individuals experiencing lower limb amputations [3]. In the United States, around $61\%$ of amputations involve at least one foot [4]. These statistics have driven research into the development of lower limb prosthetic devices, particularly focusing on the foot unit, which must efficiently absorb forces during the gait cycle, withstand body weight, and provide stability for maintaining a well-balanced posture and preventing musculoskeletal imbalances [5].

Recent advancements in materials technology have facilitated the transition from prosthetic feet suitable for individuals belonging to the K1 functional classification, typically characterized by a wooden keel, e.g. Solid Ankle Cushioned Heel (SACH) and Single Axis feet, to carbon fiber prosthetic feet with energy-storage-and-return (ESR) capabilities, associated with the K3 and K4 classifications [6, 7]. This evolution has facilitated the completion of more complex tasks, beyond simple locomotion on level surfaces at a fixed cadence, such as stair climbing and slope navigation. It has reduced walking costs [8], also expanding the opportunities to participate in sports, from recreational and rehabilitative activities to high-level competition, including the Paralympic Games, favoring an increase in societal integration among people with limb deficiencies by improving mental well-being and boosting self-confidence [9].

Although ESR feet represent an effective solution for individuals with lower-limb amputations, they do not fully reproduce the complex biomechanics and rich articulation of the human foot, and therefore still present significant limitations with important consequences for users. Their prolonged use is a major contributing factor to conditions such as osteoarthritis and osteoporosis due to musculoskeletal imbalances [5].

Other passive prosthetic devices incorporate clutch–spring arrangements to boost push-off performance ([10]), or mechanical design capable of providing both a wide ankle range of motion and an integrated system for energy storage and through the relative rotation during gait of its main four components using torsion springs [11].

In contrast, only motorized systems that add external energy are able to deliver the net positive work needed for human walking. Even so, current powered ankles typically provide only modest gains compared with ESR feet [12], and users often continue to exhibit gait asymmetries [13]. These devices also commonly suffer from drawbacks such as high cost, substantial mass and mechanical complexity. To address some of these limitations, several experimental designs integrate compliant components, placed in series or in parallel with the actuator, to lighten the drive system and enhance efficiency. These systems aim to optimize the capture of energy during stance and its release at push-off through methods such as actively regulating a locking mechanism, pre-tensioning elastic elements, or combining both strategies [14–17].

Despite advances in active ankle–foot prostheses, most of them still rely on stiff, planar carbon fiber soles, which restrict their ability to adapt to irregular terrain, reducing stability and the likelihood of falls. This remains a serious and unresolved issue for prosthetic users [18, 19], posing a major challenge to the scientific community, as highlighted in [20, 21].

In [22], we analyzed how rigid and flat soles, which do not conform to uneven surfaces, reduce the walking support area and compromise stability when encountering non-flat obstacles. Consequently, we introduced the concept of a passive adaptive prosthetic foot that more closely mimics the functionality of the human ankle-foot system. This approach has been shown to improve stability in bipedal walking, as demonstrated in [23], where the SoftFoot was introduced as a robotic foot for humanoid robots.

In this context, we developed a novel prosthetic foot, the SoftFoot Pro, inspired by the original robotic design. It features a compliant sole architecture that increases the contact surface and conforms to ground irregularities, mimicking the behavior of the human foot [22]. However, in translating the concept from robotics to prosthetic applications, it was necessary also to address the challenge of assisting the user during the terminal stance phase, when a significant effort is required for forward progression of the body — a need not present in bipedal robots with actuated joints.

To overcome this, a passive agonist–antagonist mechanism inspired by human anatomy was integrated at the ankle joint, enabling energy storage and return during gait while preserving ground adaptability [24]. This combined approach aims to reduce user fatigue, improve forward propulsion, and emulate the synergistic action of the human shank-ankle-foot complex. With the SoftFoot Pro, we successfully participated in the Cybathlon 2024 Leg Prosthesis Race, achieving second place. As is well known, the Cybathlon is an international competition where individuals with physical disabilities compete using advanced assistive technologies. In the Leg Prosthesis Race, pilots perform a series of everyday mobility tasks designed to test balance, adaptability, and overall prosthetic functionality, closely reflecting real-world challenges [25, 26].

This study evaluates the kinematic and metabolic performance of the novel agonist–antagonist SoftFoot Pro (combining energy storage and adaptive sole features) in comparison to commercial carbon fiber feet (energy storage only) and the original SoftFoot (adaptive sole only). User impressions and experiences were also assessed. The analysis was conducted with the official pilot of the SoftFoot Pro team during a dedicated pre-competition training session. The pilot completed the full Cybathlon Leg Prosthesis Race track twice consecutively. An in-depth kinematic analysis was carried out for both the level walking and stair-walking tasks of the Cybathlon, focusing on lower-limb joint range of motion and providing a detailed description of joint angle patterns in the sagittal plane. Gait symmetry and walking speed were also evaluated across the three prosthetic feet. In addition, a more general kinematic assessment was performed by analyzing the maximum angular excursions at each lower-limb joint. The comprehensive kinematic and metabolic assessments demonstrated that the agonist–antagonist SoftFoot Pro significantly improved performance compared to the original SoftFoot across a wide range of demanding locomotor tasks requiring functional capabilities beyond simple level-ground ambulation, while achieving similar performance to the commercial carbon fiber foot. Specifically, it resulted in a reduced net metabolic cost of walking — comparable to that of the carbon fiber foot — and an increased ankle joint range of motion relative to both the original SoftFoot and the carbon fiber foot. Finally, the novel SoftFoot Pro showed the fastest track completion time and robust performance across diverse tasks. Questionnaire results further confirmed the pilot’s positive perception of stability, comfort, and overall usability, even with respect to the carbon fiber foot.

This single-subject case study provided a quantitative evaluation of novel adaptive prosthetic feet during Cybathlon tasks, which closely replicate everyday activities. Our findings suggest that integrating an ankle joint with an agonist–antagonist energy recycling system offers substantial benefits for users in real-world applications, achieving performance comparable to commercial carbon fiber feet while preserving the advantages of an adaptive sole. The Cybathlon thus served as a crucial catalyst for advancing both the innovation and validation of our adaptive prosthesis.

## Methods

### Participant

The participant, an official pilot in the Cybathlon 2024 Leg Prosthesis Race, was a 56-year-old male, 1.74 m tall and weighing 75 kg. He had a unilateral transfemoral amputation on the right side resulting from an accident at the age of 47 and belonged to the K3-K4 functional classification. During the training and the competition, the pilot wore a semi-active knee system, the Genium by Ottobock (Duderstadt, Germany), the same prosthetic knee he wore daily. The carbon fiber foot used in the experimental session was the Triton by Ottobock (Duderstadt, Germany), the commercially available passive ESR foot that the user wore daily.

To provide a visual reference of typical joint behavior in the absence of prosthetic-related issues, the performance of an unimpaired (non-amputee) subject (26 years old, 1.80 m tall, 76 kg, male) was recorded with the same set-up and condition during two tasks: level walking and stair ascending-descending.

Testing was conducted after the subjects were fully informed about the study’s aims and procedures and provided written informed consent to the experimental activity. The study was approved by the Bioethical Committee of the University of Pisa (resolution no. 48/2024).

### Experimental setup

#### SoftFootPro

**Fig. 1:**
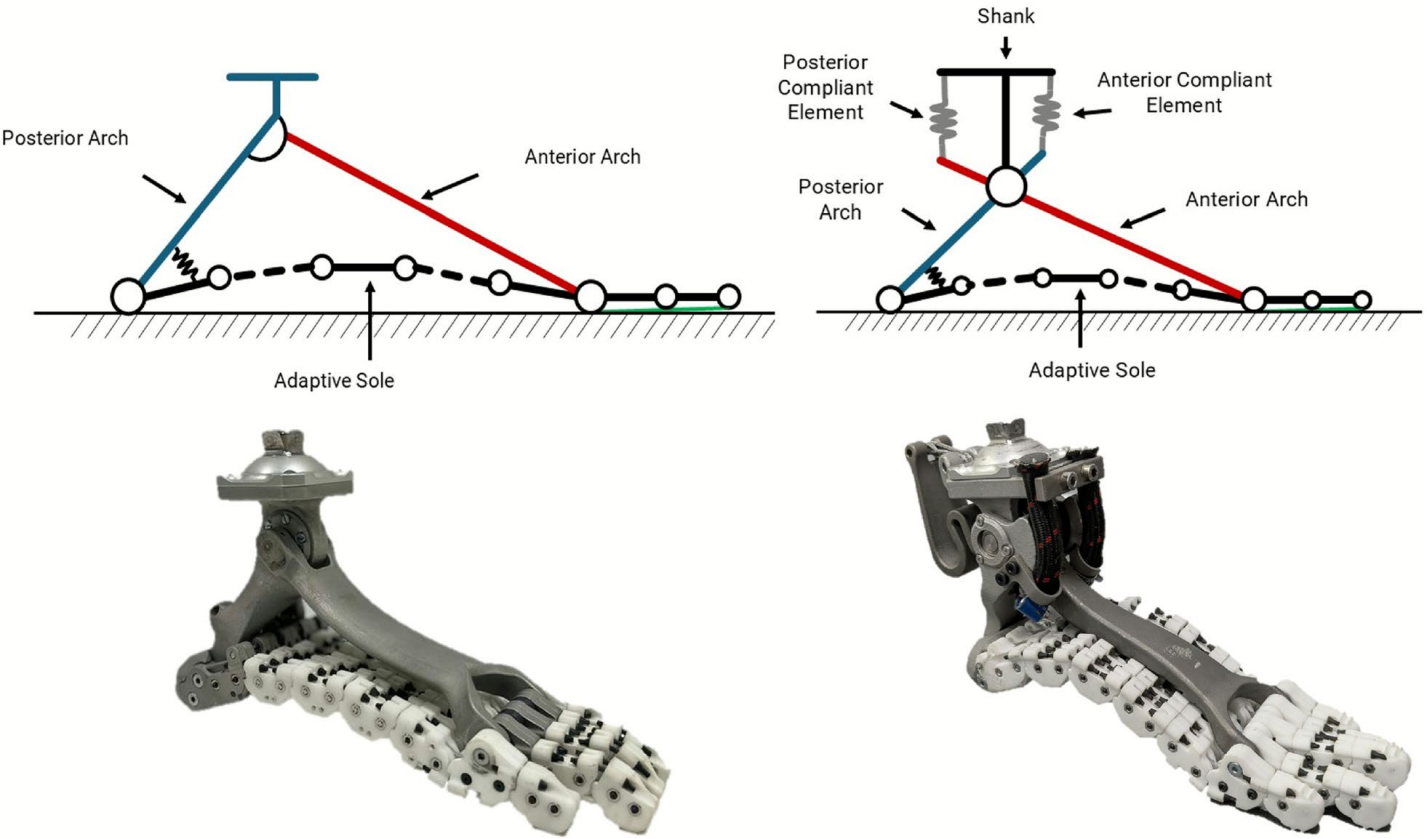
SoftFoot models and prototypes. The top row shows, on the left, the model of the original SoftFoot and, on the right, that of the agonist–antagonist SoftFoot Pro used at Cybathlon. The bottom row shows the corresponding prototypes

The SoftFoot (Fig. 1, left panels) is a passive, underactuated, anthropomorphic, and adaptive foot inspired by the complex architecture and functions of the human foot [22]. Its design aims to reproduce the ability of the human foot to adapt to the different grounds we encounter daily with its articulated structure of bones, tendons, and muscles, and its ability to stiffen to act as a rigid lever when propelling the body forward [27].

The SoftFoot compliance is determined by the underactuated sole, designed to provide adaptability on uneven terrains. The sole features five flexible parallel plantar modules, each made of smaller units coupled with rolling contact joints [28], going from heel to toe. Adjacent units are connected with elastic bands, and an inextensible tendon routes following a specific path through each module, giving the foot a stress-stiffening behavior. In this way, the structure is compliant under low loads, and it stiffens under higher loads, effectively supporting the user’s weight throughout the gait cycle. Two rigid elements, the rear and the frontal arches, connect the sole to the user’s pilon.

The SoftFoot Pro, shown on the right side of Fig. 1, improves the original SoftFoot by incorporating a passive agonist–antagonist ankle mechanism that enables the system to store and release energy in response to the user’s load throughout the gait cycle. This mechanism introduces an anterior and a posterior spring set, with respect to the ankle joint, connecting the arches and the shank. The shank link (AlSi10Mg) is free to rotate about the ankle joint and is connected to the pilon, enabling a more natural ankle motion and effectively transmitting the mechanical action of the springs to the user. The anterior spring set, two elastic bungee cords (from Sandow Technic, Germany), replicates the function of the dorsiflexor muscles in human walking, aiding in shock absorption during the loading response phase. Conversely, the posterior spring, a customized S-shaped titanium spring (Ti6Al4V), mimics the behavior of the Achilles tendon and plantarflexor muscles, reducing energy dissipation and thereby enhancing walking efficiency. This prototype represents a redesigned version of the device presented in [24], specifically developed for the Cybathlon competition. This updated design aims to achieve a more compact and user-friendly solution, keeping the same core architecture but with a more functional physical implementation to make it more suitable for everyday use and for the competition.

#### Data acquisition

Lower-limb kinematics were recorded using a portable, wireless, inertial kinematic measurement system (Xsens MVN Awinda system, Movella, Henderson, USA). This system was preferred over traditional optical motion capture systems, which require the placement of predefined markers that could be displaced during the dynamic activities and demand specific environmental conditions for camera calibration and optimal functionality [29]. Marker occlusions also pose a significant limitation in such setups.

**Fig. 2:**
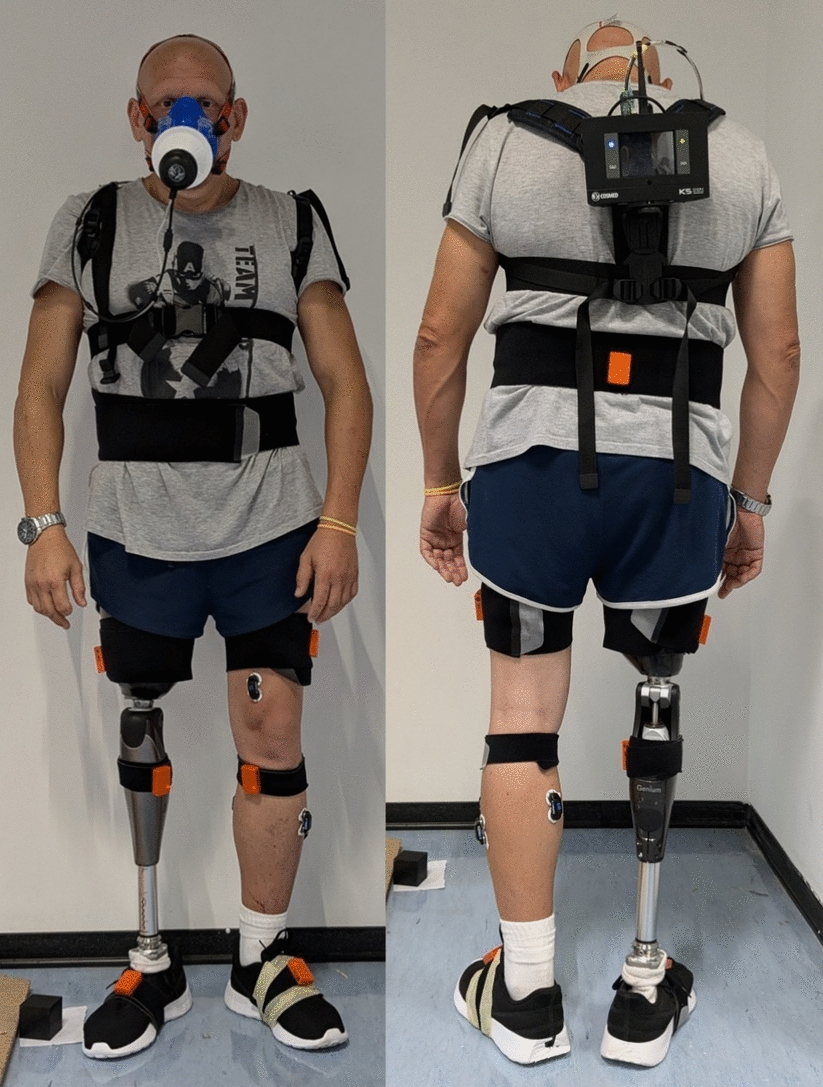
The figure shows frontal and rear views of the subject equipped with all the sensors: IMU in orange and the K5 metabolic analyzer

Kinematic data were collected at 100 Hz using 7 Inertial Measurement Units (IMU) (dimensions: 47 mm × 30 mm × 13 mm; weight: 20 g) positioned on the feet, lower legs, upper legs, and pelvis, following the Xsens setup guidelines (see Fig. 2) [30, 31]. Before data acquisition, the subject’s body measurements were registered and set on the system software, and a sensor-to-segment calibration was performed while the participant stood in a predefined pose (i.e., N-pose, with the arms along the trunk). The acquisition scenario was configured to “Multilevel” mode in the Xsens software to account for the subject’s interaction with flat and varying-height terrains.

The metabolic data were recorded with the Cosmed K5 (COSMED, Italy), a portable metabolic analyzer used to measure respiratory gases and assess energy expenditure during rest or physical activity in clinical, sports, and research settings. The calibration process included flowmeter calibration, scrubber reset, and reference gas calibration to compensate for potential environmental factors affecting turbine accuracy, to zero the CO_2_ analyzer, and to set gas concentrations to known reference values [32]. A face mask, equipped with the flowmeter, was securely fitted to the subject using adjustable elastic bands and a harness, supporting the main unit of the system, weighing less than 1 kg, positioned on the back of the user, as in Fig. 2. The system operated in Telemetry Data Transmission mode, transmitting data wirelessly to a dedicated PC station.

#### Leg prosthesis race

**Fig. 3:**
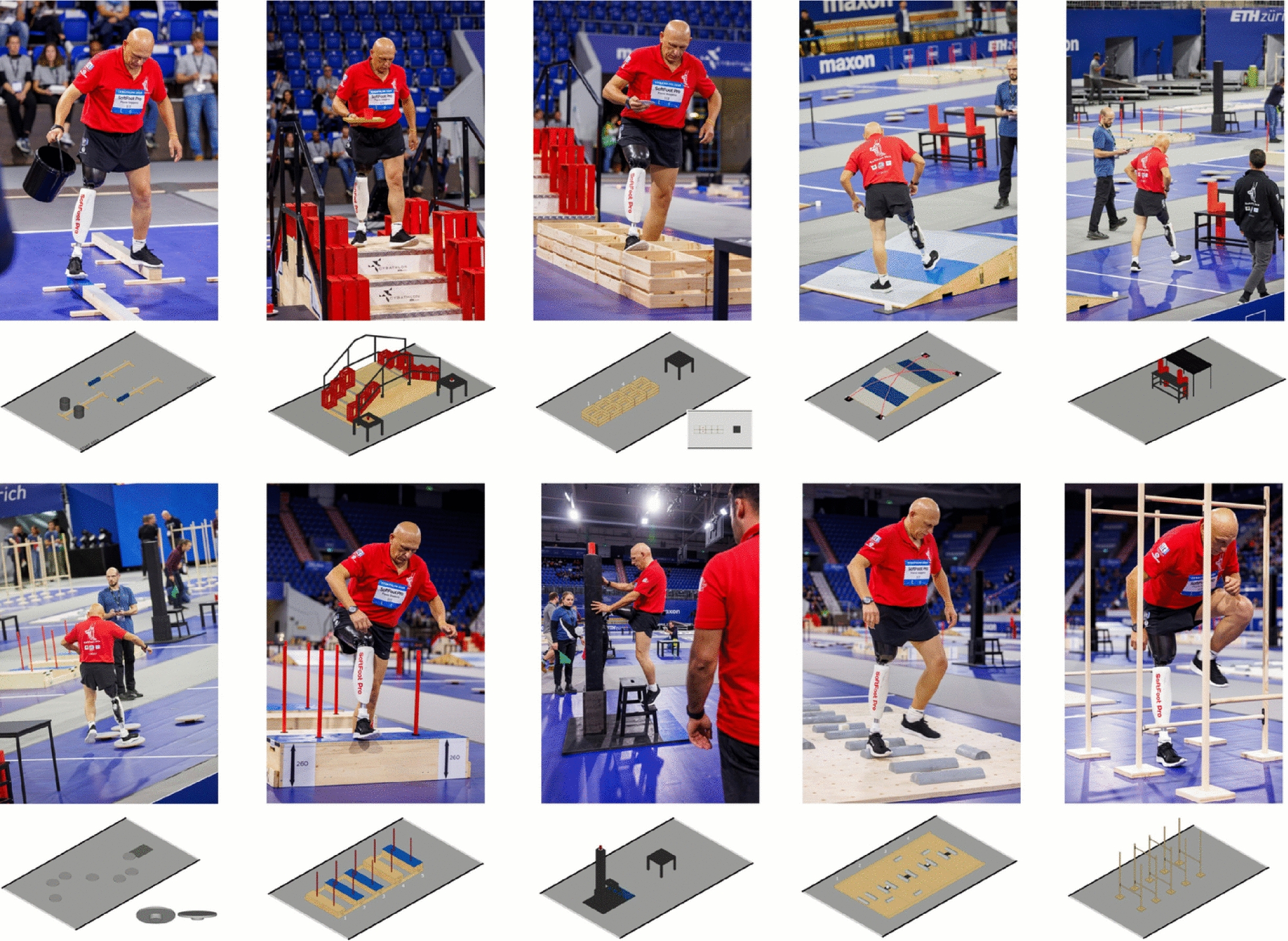
Cybathlon 2024: Tasks. The figure shows the ten Cybathlon 2024 tasks as performed by the SoftFoot Pro team pilot during the final, along with the setup of each task. The top row, from left to right, shows the Balance beam, Stairs, Step over, Slopes and Bench and table tasks. The bottom row, from left to right, shows the Wobbly steps, High step, Ladder, Cross country and Hurdles tasks

The race consisted of ten tasks designed to simulate everyday challenges faced by individuals with lower limb loss. They are presented in the order in which they were completed during the Zurich competition in Fig. 3. We reproduced the setup in our laboratory according to the instructions for the materials and equipment reported in [26]. A more detailed description of the tasks and the rules for their invalidation can be found at [26].

An additional video file demonstrates the fitting process of the SoftFoot prosthesis, its integration into daily activities, and its application during both training and the official competition (see Supplementary video).

### Experimental protocol

Data collection was conducted in a spacious laboratory room set up to replicate all tasks from the official Leg Race discipline of the Cybathlon 2024 event; no other wireless devices were present that could interfere with the data recording. Initially, the calibration procedures required for the Cosmed K5 (flowmeter calibration, scrubber reset, and reference gas calibration) were completed. The subject was familiarized with the measurement devices and then equipped with the IMU sensors. Once the IMUs calibration phase was performed, the subject was equipped with the Cosmed K5. The subject was asked to complete two consecutive rounds of the Cybathlon circuit following the competition task order at a competition-like pace with three different prosthetic feet: the Triton foot, the SoftFoot Pro, and the original SoftFoot, in this exact order. The prosthetic feet were worn inside a running shoe (KLNJ BE ESSENTIAL, Decathlon), that was used both during the experiments and in the official Cybathlon competition. The shoe’s sole was flexible enough to accommodate the bending of the SoftFoot sole, while remaining sufficiently stiff to prevent the toes or heel of the SoftFoot from contacting the ground during the cross-country task, which would have invalidated the task. Two rounds were required to make the trial long enough to reach a metabolic steady state condition. No familiarization phase was required on the test day for the prosthetic devices, as the subject had already become extensively familiar with them during multiple training sessions in the weeks leading up to the test day. The experiment continued even if errors occurred that would have invalidated a specific task. For each tested foot, the subject was initially asked to rest for 5 min on a chair to acquire resting metabolic data. He then completed two rounds of the Cybathlon circuit without breaks and finally performed three repetitions of walking on level ground along a seven-meter path. A five-minute break was provided to change the prosthetic foot. Afterwards, a two-hour period of inactivity followed before restarting the protocol. During this time, the participant was asked to complete four standardized questionnaires to provide qualitative feedback on the prosthetic foot tested.

The questionnaires included:

- **Prosthetic Evaluation Questionnaire (PEQ)**: Comprising nine validated multi-item scales, plus additional individual questions. Mobility was assessed using the Locomotion scale (8 questions) and five questions under the Transfer category [33].
- **Locomotor Capabilities Index (LCI-5)**: A self-reported measure of prosthetic mobility, designed to assess walking ability. It uses five response levels (0 to 4), with the original third level divided into two categories for greater detail [gailey:2012], [34].
- **System Usability Scale (SUS)**: A five-point Likert scale that measures the perceived usability of each prosthetic foot [35, 36].
- **NASA Task Load Index (NASA-TLX)**: A five-point Likert scale used to evaluate perceived cognitive and physical workload across multiple independent dimensions [37, 38].

### Data analysis

During preliminary trials, in Task 5, “Bench and Table”, the subject accidentally bumped the table while sitting on the bench, due to the small distance between them, causing an undesired change in the position of IMU sensors, thus altering data acquisition. Therefore, this task was excluded from the official testing session, and the analysis presented in this study includes only nine of the ten Cybathlon 2024 Leg Race tasks. Kinematic and metabolic analyses were conducted using MATLAB R2023b.

#### Kinematic analysis

Hip, knee, and ankle raw signals were extracted from both the intact limb (IL) and the prosthetic limb (PL). The analysis was conducted across three key domains: level walking, stair ascent and descent, and the complete set of nine Cybathlon tasks. All kinematic and spatio-temporal parameters described below were compared across the three tested prosthetic feet. As the data were collected from a single subject, comparisons were made for visual inspection only, and no statistical analysis was performed.

##### Level walking task

Gait cycles immediately preceding or following a change in walking direction were excluded to avoid transitional effects. Ten gait cycles per limb were time-normalized to express each angular trajectory as a percentage of the gait cycle. Each angular signal was resampled to yield 150 data points uniformly distributed across the gait cycle. For each joint and limb, the mean and standard deviation of the angular trajectories were computed and compared across the three types of prosthetic feet. Additionally, the kinematic analysis included calculation of joint range of motion (ROM) — defined as the average across gait cycles of the difference between the maximum and minimum joint angles within each cycle. Mean stride length and gait speed were also computed for each prosthetic foot condition, averaged over four consecutive gait cycles, selected to exclude steps involving a change of walking direction, and normalized to the subject’s body height (BH).

##### Stairs task

Following the extraction of raw angular signals, the central gait cycles were selected for analysis. Specifically, three gait cycles were analyzed during ascent and three during descent for each limb, corresponding to the middle steps of the ramp. Each angular trajectory was time-normalized and resampled to 150 data points, representing 0–100% of the gait cycle. An analysis of the transition steps from “walk to stair ascent” and from “stair descent to walk” was conducted for completeness, and the resulting joint kinematics is presented in Supplementary results.

##### Cybathlon tasks

For all Cybathlon tasks, the maximum angular excursion was obtained as the difference between the maximum and minimum joint angle values recorded during the entire execution of each task. The completion time of each task and the total time for the circuit were obtained directly from the Xsens software.

#### Metabolic analysis

For the metabolic analysis, six minutes were necessary to reach the metabolic plateau after the transient phase, allowing for an accurate examination of the subject’s metabolic response under exertion. For this reason, two consecutive rounds of the Cybathlon race were performed.

The rate of oxygen consumption (VO_2_) and the Respiratory Exchange Ratio (RER), provided by the Cosmed K5 system as the ratio between carbon dioxide produced to oxygen consumed, were extracted during both the resting phase and the activity plateau phase. VO_2_ data were normalized to the subject’s body mass, and mean values were computed for both the resting phase and the second round of the Cybathlon. Subsequently, the Net Cost of Transport (NCoT) was calculated for each of the three prosthetic foot designs to compare the metabolic demands required to complete the activities using the following equation:

1 $$\begin{aligned} NCoT = \frac{EEq-RMR}{WS \times 60}. \end{aligned}$$

where EEq is the Energetic Equivalent of the oxygen consumed in [*J*/*kg*/*min*], RMR is the Resting Metabolic Rate, i.e., the energy consumption at rest [*J*/*kg*/*min*], and WS is the walking speed [*m*/*s*] [39]. The EEq is calculated as:

2 $$\begin{aligned} \text {EEq} = (15962 + 5155 \times \text {RER}_{cyb}) \left( \frac{\text {VO}_2{}_{cyb}}{1000} \right) \end{aligned}$$

where $\text {RER}_{cyb}$ is the mean value of the RER during the Cybathlon second round, and $\text {VO}_2{}_{cyb}$ is the mean value of the $\text {VO}_2$ during the Cybathlon second round. While the RMR is defined as:

3 $$\begin{aligned} \text {RMR} = (15962 + 5155 \times {RER}_{rest}) \left( \frac{{VO}_2{{rest}}}{1000} \right) \end{aligned}$$

where $\text {RER}_{rest}$ is the mean value of the RER during the resting phase, and $\text {VO}_2{}_{rest}$ is the mean value of the $\text {VO}_2$ during the resting phase. Finally, Heart Rate (HR) was also monitored throughout the activity to provide complementary data on cardiovascular effort.

#### Questionnaires and cybathlon performance

The pilot was asked to complete four questionnaires, as previously mentioned in 2.3.

The first one was the PEQ, consisting of 82 items. The participant rated each of the three prosthetic feet using a 100-*mm* analog scale. Scale scores were calculated as the arithmetic mean of all answered items within each scale, provided that at least half of the items were completed, and then expressed on a 0–100 scale.

The second questionnaire was the LCI-5, comprising 14 items, scored from 0 to 4 (0 = no; 1 = yes, with help; 2 = yes, with supervision; 3 = yes, alone with aids; 4 = yes, alone without aids). Basic and advanced mobility subscale scores were computed separately by summing the responses to their respective 7 items, each expressed out of a maximum of 28 points. The total LCI-5 score was obtained by summing these subscale scores and reported out of 56.

The SUS and NASA-TLX questionnaires were also administered to evaluate perceived usability and workload for each prosthetic foot. For the SUS, the participant rated 10 items (R_n_, with $n = 1, 2, \dots , 10$) on a 5-point Likert scale (1 = “strongly disagree”, 5 = “strongly agree”). Scores were then recalculated following [36]. For odd-numbered items, the adjusted scores were computed as $Q_{\text {i}} = R_{\text {i}} - 1$, ($i = 1,3,5,7,9$), while for even-numbered items they were calculated as $Q_{\text {k}} = 5 - R_{\text {k}}$, ($k = 2,4,6,8,10$). The final SUS score (SUS_score_) was then expressed out of 100 by the equation:

4 $$\begin{aligned} SUS_{\text {score}} = (\sum _{n=1}^{10} Q_{\text {n}}) \times 2.5 \end{aligned}$$

The six subscales of the NASA-TLX were rated on a 5-point Likert scale (1 to 5), with results visualized using radar charts.

In addition to questionnaire data, a performance analysis of the final race of the 2024 Cybathlon Leg Race final was conducted by comparing task completion times and success rates across the four finalist teams. User feedback on both the original SoftFoot and the SoftFoot Pro was collected during training and post-competition debriefings.

## Results

### Kinematics

#### Level walking

**Fig. 4:**
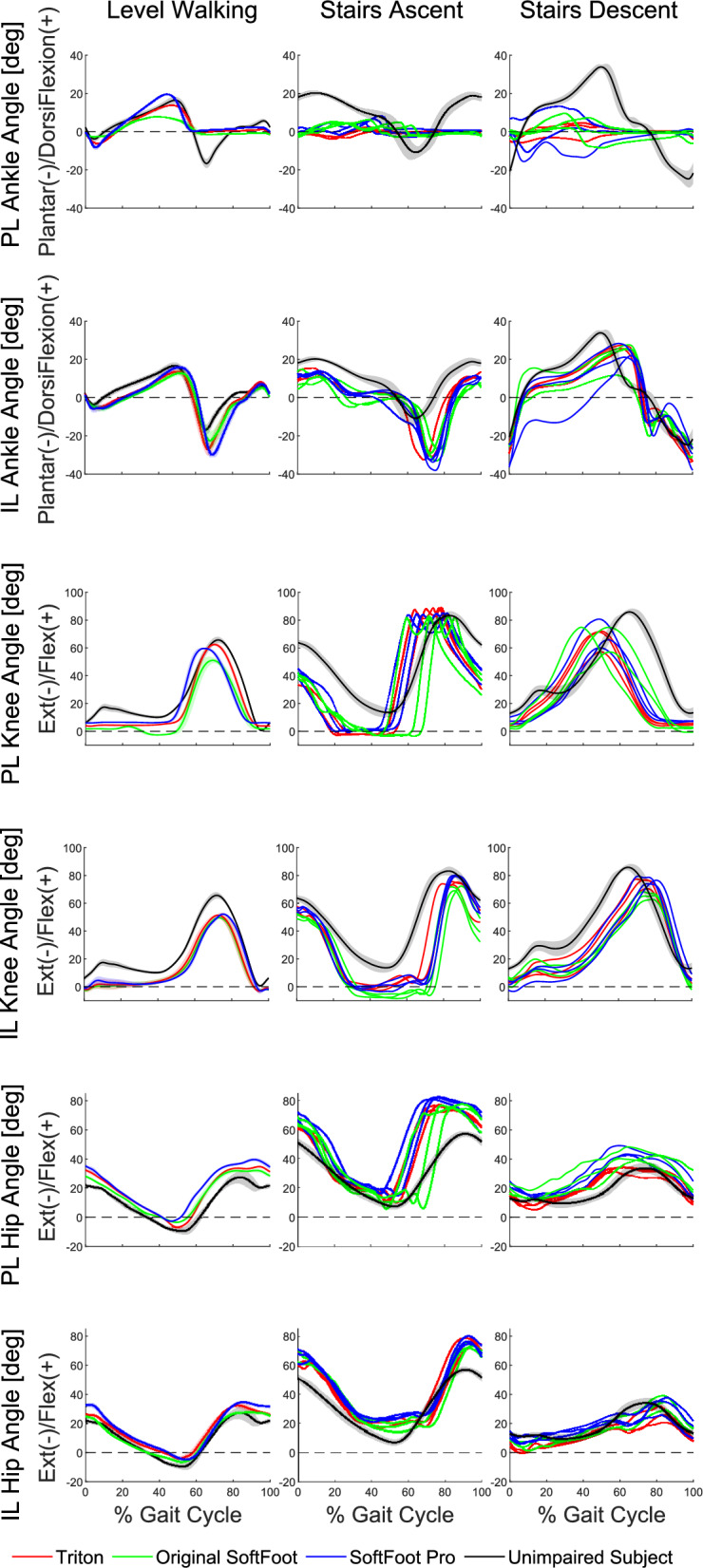
Sagittal plane ankle, knee and hip angle patterns of the prosthetic limb (PL) and intact limb (IL). From left to right, level walking (mean value from 10 strides), stairs ascent (3 steps), and stairs descent (3 steps) angles are reported for the Cybathlon pilot using the three different feet: Triton (red), original SoftFoot (green), and SoftFoot Pro (blue). The joint angles of an unimpaired subject are shown for comparison (black)

**Fig. 5:**
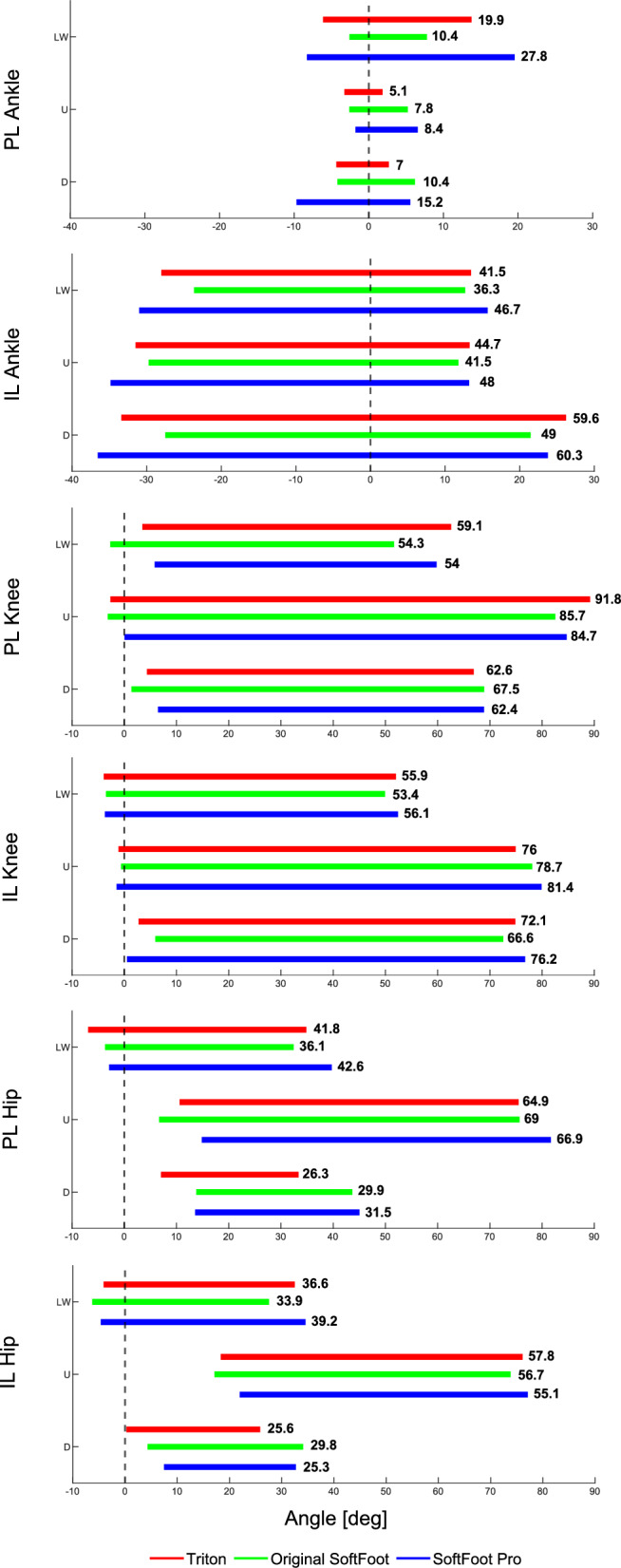
Color-coded bars (Triton (red), original SoftFoot (green), and SoftFoot Pro (blue)) represent ankle, knee, and hip ROM of the Cybathlon pilot’s prosthetic limb and intact limb during level walking (LW), stairs ascent (U), and stairs descent (D). Mean ROM values are also reported next to the corresponding bar

Figure 4 shows the sagittal plane joint angle patterns (ankle, knee, and hip) for the prosthetic limb and the intact limb during level walking, stair ascent, and stair descent. The mean range of motion values of the same joints are presented in Fig. 5.

During level walking, the biological ankle exhibited average plantarflexion and dorsiflexion peaks of approximately $4^{\circ }$ and $16^{\circ }$, respectively, during the early and late stance phases. In the swing phase ($60\%$ - $100\%$ of the gait cycle), the average plantarflexion peak reached approximately $16^{\circ }$. The knee exhibited average flexion peak of approximately $18^{\circ }$ during the stance phase and $65^{\circ }$ during the swing phase.

During early stance, the subject’s prosthetic ankle reached an average plantarflexion peak of $2^{\circ }$ with the original Softfoot, $6^{\circ }$ with Triton, and $8^{\circ }$ when exploiting the Softfoot Pro. At the end of the stance, the mean value of dorsiflexion peak was $8^{\circ }$ with the original Softfoot, $13^{\circ }$ with Triton, and $19^{\circ }$ with the Softfoot Pro. All three feet showed no plantarflexion movement during the swing phase. At the sound ankle, the average plantarflexion peak in early stance was $6^{\circ }$ across all prosthetic feet, while the average dorsiflexion peak in late stance was $13^{\circ }$ with the original SoftFoot and Triton, and increased to $15^{\circ }$ with the SoftFoot Pro. Furthermore, the Triton and the SoftFoot Pro exhibited greater peak plantarflexion at mid-swing, with average values of $27^{\circ }$ and $29^{\circ }$, respectively, while the original SoftFoot reached only $21^{\circ }$.

The knee joint angle in the prosthetic limb exhibited a flat pattern throughout the entire stance phase when using the Triton and SoftFoot Pro, whereas with the original SoftFoot this flat pattern, observed only in $0\%$ - $20\%$ of the gait cycle, was followed by a prolonged hyperextension during late stance ($30\%$ - $50\%$ of the gait cycle), just before the flexion peak in the swing phase, with an average hyperextension peak of $3^{\circ }$. In the intact limb, the early stance flexion peak ($12\%$ of the gait cycle) was absent when using the original SoftFoot and Triton, unlike the SoftFoot Pro (average peak of $6^{\circ }$).

The prosthetic hip remained consistently more flexed than that of the unimpaired subject.

From the ROM analysis emerged that the prosthetic ankle achieved an average ROM of $27.8^{\circ }$ when the Softfoot Pro was used, which was about $40\%$ higher than the Triton ($19.9^{\circ }$) and approximately $167\%$ greater than the original SoftFoot ($10.4^{\circ }$). At the knee and hip joints of both the prosthetic and the intact limb, differences between ROM values were less marked across different prosthetic feet.

Finally, Table 1 reports the mean and standard deviation values for stride length and gait speed during level walking, normalized to the subject’s height and expressed as percentages, for both the Cybathlon pilot and the unimpaired subject. The unimpaired subject exhibited an average stride length value of 1.2 m and an average walking speed of 0.92 m/s. Stride length values were comparable between the prosthetic limb and the intact limb across all three prosthetic feet. The Triton and the SoftFoot Pro feet showed an average stride length of 1.2 m for both the prosthetic and intact limbs. The original SoftFoot resulted in a stride length of 1.0 m. The average walking speeds, expressed in m/s, were 0.95 for the Triton and the SoftFoot Pro, 0.75 for the original SoftFoot.

**Table 1:** Mean and standard deviation values for stride length (PL = prosthetic limb; IL = intact limb) and average gait velocity (normalized to body height and expressed as a percentage) during level walking. Data are reported for the Cybathlon pilot using the three tested prosthetic feet, as well as for the unimpaired subject

Subjects	Prosthetic Foot	Stride Length/BH [%]		Gait Speed/BH [% $s^{-1}$]
		PL	IL	
Pilot	Triton	**68**.**2** $\pm$ **4**.**0**	**69**.**3** $\pm$ **1**.**5**	**54**.**5** $\pm$ **3**.**7**
	Original SoftFoot	60.1 $\pm$ 1.6	60.3 $\pm$ 1.2	43.2 $\pm$ 2.6
	SoftFoot Pro	**71**.**0** $\pm$ **3**.**7**	**70**.**7** $\pm$ **2**.**7**	**54**.**3** $\pm$ **5**.**4**
Unimpaired subject		69.2 $\pm$ 2.2		52.7 $\pm$ 1.9

Among the different prosthetic feet, closest values for stride length and gait speed are highlighted in boldfont

#### Stairs task

During stair ascent, the biological ankle began with $20^{\circ }$ of dorsiflexion and reached a peak plantarflexion of approximately $10^{\circ }$ at the end of the stance phase. The knee and hip exhibited a similar pattern: they started with approximately 60° and 50° of flexion, respectively, followed by an extension movement during the stance phase and a subsequent flexion during the swing phase. The main difference in the transition phases (Supplementary results) was observed at the left ankle during the transition from stair descent to walk, where no plantarflexion occurred at the end of the swing phase.

With all three prosthetic feet, the prosthetic ankle maintained a neutral position during both the loading response and swing phases, showing limited dorsiflexion motion at the end of the stance, with different patterns observed across the three prosthetic feet. The prosthetic knee started with approximately $40^{\circ }$ of flexion, with minimal variation across feet and steps, and remained in a more extended position throughout the stance phase, reaching a neutral configuration. The prosthetic hip remained consistently more flexed than the biological hip, especially at initial contact and during terminal swing.

In the transition steps from walk to stair ascent (Supplementary results), the participant’s prosthetic ankle showed similar patterns to those observed for the ascent central step analysis, while the prosthetic knee remained fully extended (0°) throughout the whole stance phase, with minimal variation across feet and steps. The prosthetic hip started with much smaller flexion angles compared to the corresponding angles observed in stair ascent central-step analysis.

During stair descent, the unimpaired subject initiated the gait cycle with approximately $20^{\circ }$ of plantarflexion and reached a peak dorsiflexion of about $30^{\circ }$ during the stance phase. In the 0% and 40% interval of the gait cycle, the prosthetic ankle exhibited reduced dorsiflexion, with peak values varying depending on the prosthetic foot. During the swing phase, the prosthetic ankle returned to a neutral position across all three prosthetic feet, without showing plantarflexion. The biological ankle reached $20^{\circ }$ of plantarflexion to initiate the subsequent step. The unimpaired subject showed greater knee flexion, while the Cybathlon pilot exhibited increased hip flexion.

In the transition steps from stair descent to walk (Supplementary results), the participant’s prosthetic ankle showed approximately 30° of plantarflexion between 10% and 40% of the gait cycle in two of the three trials when using the SoftFoot Pro. Ankle angle profiles were similar to those observed for the central step of the stair descent when using the Triton and the original SoftFoot. All three prosthetic feet showed knee and hip angle profiles for the prosthetic limb similar to those observed for the central step of the stair descent.

Joint angle patterns were generally similar between the participant’s intact limb and the unimpaired subject, with the participant’s sound limb exhibiting a greater overall range of motion.

The analysis of the range of motion revealed that in the stair ascent task, the SoftFoot Pro achieved a ROM of $8.4^{\circ }$, representing a $65\%$ increase over the Triton ($5.1^{\circ }$) and an $8\%$ increase over the original SoftFoot ($7.8^{\circ }$). In stairs descent, the SoftFoot Pro reached a ROM value of $15.2^{\circ }$, marking a $117\%$ increase compared to the Triton ($7.0^{\circ }$) and a $46\%$ increase over the original SoftFoot ($10.4^{\circ }$). The knee and hip joints of both the prosthetic and intact limbs exhibit comparable ranges of motion.

#### Cybathlon tasks: maximum angular excursion

For all the Cybathlon’s tasks of the Leg Race performed during the circuit, an overall assessment of lower limb kinematics was conducted by evaluating the maximum angular excursion for the ankle, knee, and hip joints for both limbs.

**Fig. 6:**
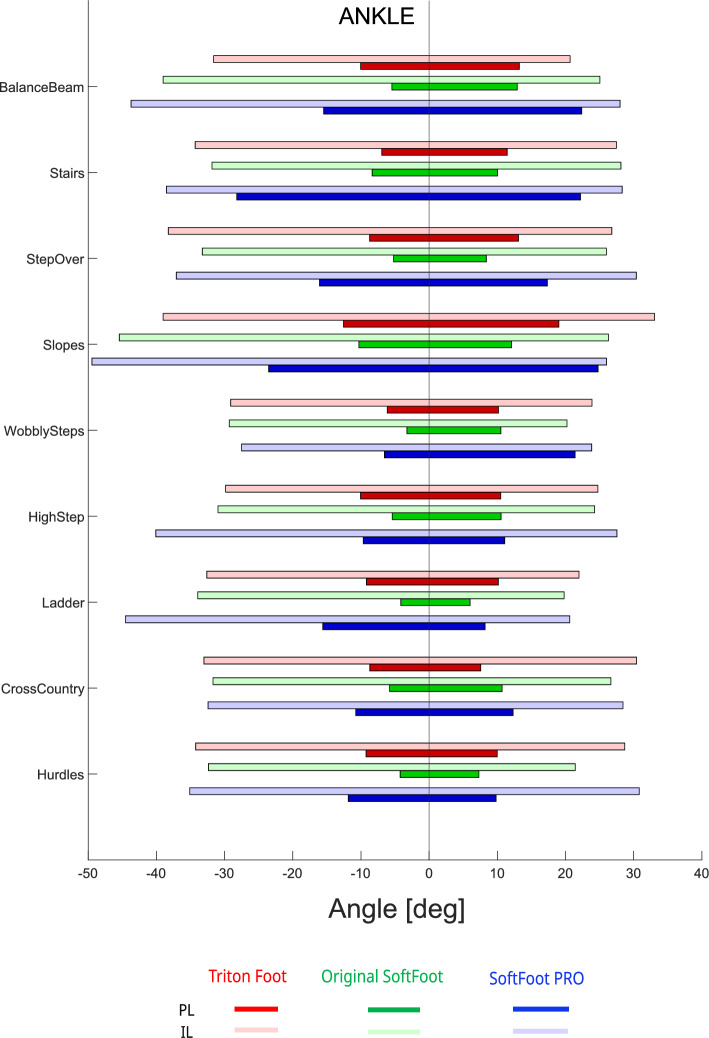
Angular excursion at the ankle joint of the prosthetic limb (PL, dark bars) and the intact limb (IL, light bars) during all Cybathlon tasks performed by the pilot. Different prosthetic feet are represented by color-coded bars: Triton (red), original SoftFoot (green), and SoftFoot Pro (blue)

At the ankle joint (Fig. 6), the angular excursion of the prosthetic ankle was the highest when using the SoftFoot Pro across most tasks; the only exceptions were during the High Step and Cross Country tasks. Conversely, the original SoftFoot exhibited the lowest angular excursion across all tasks, except for the Cross Country task, where its amplitude was comparable to both the Triton foot and the SoftFoot Pro, and the Stairs task, where it was similar to the Triton foot.

**Fig. 7:**
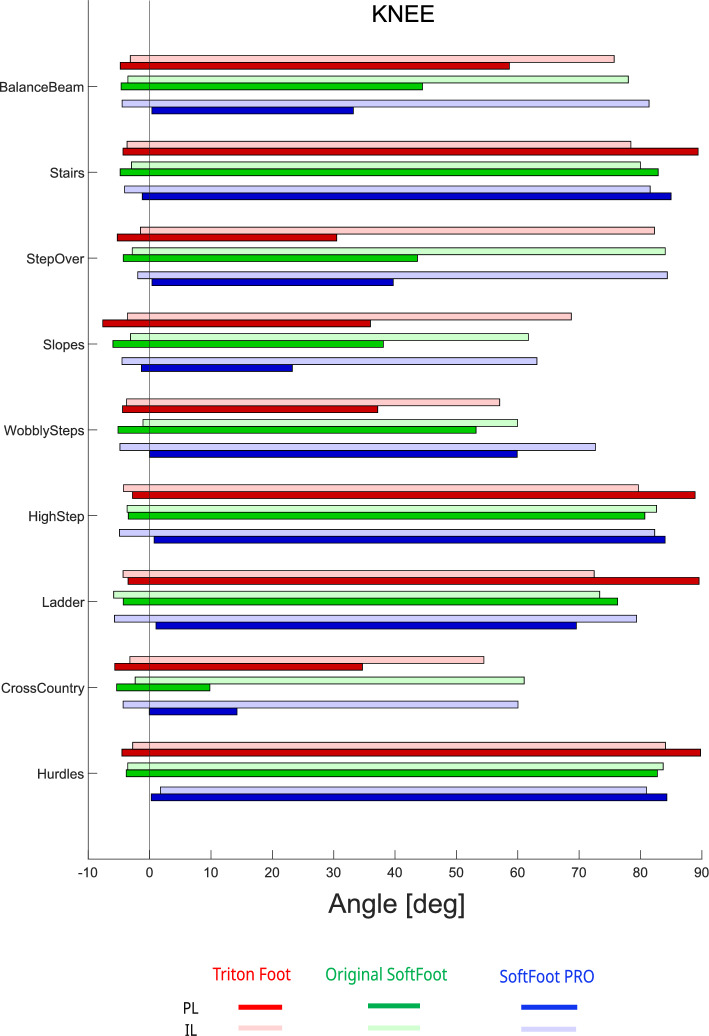
Angular excursion at the knee joint of the prosthetic limb (PL, dark bars) and the intact limb (IL, light bars) during all Cybathlon tasks performed by the pilot. Different prosthetic feet are represented by color-coded bars: Triton (red), original SoftFoot (green), and SoftFoot Pro (blue)

At the knee joint (Fig. 7), the greatest angular excursions were required for Stairs, High Step, Ladder, and Hurdles tasks, while Cross Country, Wobbly Steps, and Slopes elicited the smallest amplitude of motion. The prosthetic knee achieved the highest flexion in 6 out of the 9 tasks, reaching approximately 90 degrees in four of them—Hurdles, Ladder, High Step, and Stairs—when using the Triton foot. The other two feet showed overall reduced knee flexion in these tasks compared to the Triton foot, but greater knee flexion during Wobbly Steps and Step Over.

**Fig. 8:**
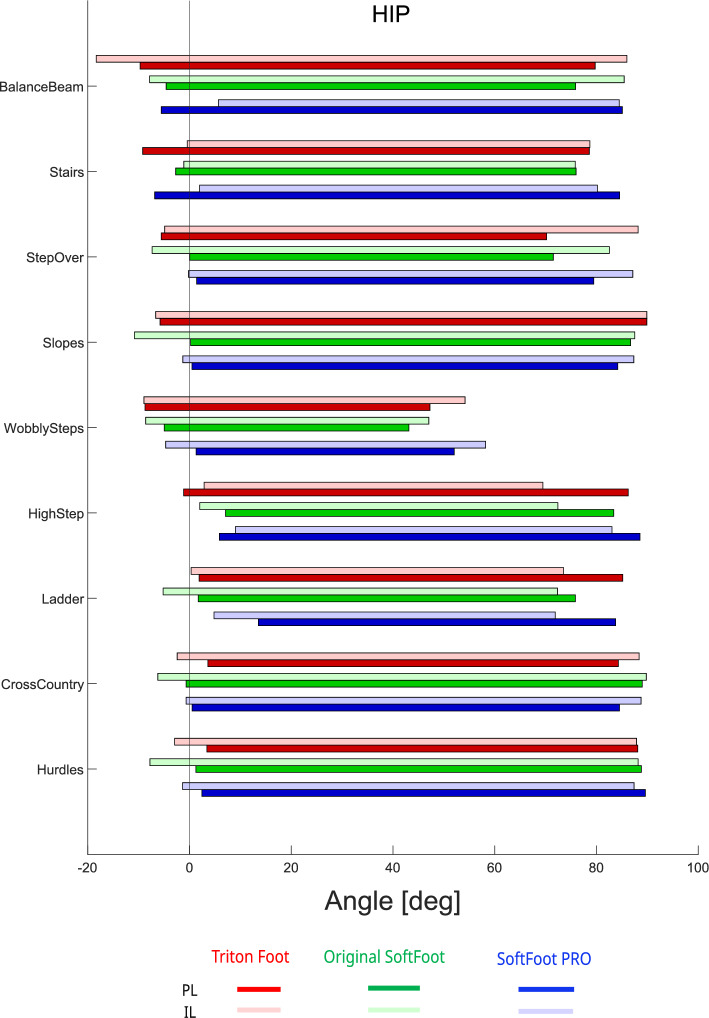
Angular excursion at the hip joint of the prosthetic limb (PL, dark bars) and the intact limb (IL, light bars) during all Cybathlon tasks performed by the pilot. Different prosthetic feet are represented by color-coded bars: Triton (red), original SoftFoot (green), and SoftFoot Pro (blue)

At the hip joint (Fig. 8), the most notable result was the high degree of flexion observed in both the sound and prosthetic limbs, across all three prosthetic feet and in all tasks —except for the Wobbly Step, which showed flexion values more comparable to those typically seen during level walking.

### Task performance: completion times

The times required to complete each task, as well as the total time for the second lap of the circuit, are reported in Table 2. The SoftFoot Pro was the fastest in six out of nine tasks, achieving a total completion time of 290 s. The overall completion times were 295 s for the original SoftFoot and 310 s for the Triton.

**Table 2:** Completion times in seconds for the nine Cybathlon tasks and the overall second circuit lap for each of the three tested prosthetic feet

Cybathlon tasks	Prosthetic feet		
	Triton [s]	Original SoftFoot [s]	SoftFoot Pro [s]
Balance Beam	30.1	32.7	29.3
Stairs	33.5	33.2	34.8
Step Over	42.7	44.7	35.1
Slopes	27	26	24.2
Wobbly Steps	8.3	9.5	8.2
High Step	24.2	20.9	19.5
Ladder	25.3	18.1	18.8
Cross Country	24	20	29.1
Hurdles	40.8	36	35.5
Total	310	295	290

### Metabolic cost

Table 3 presents the main metabolic parameters calculated for the second circuit lap with the three feet.

Specifically, the VO_2__plateau_ values were 18.8, 19.9, and 18.7 [mL/(kg*min)] for the Triton, the original SoftFoot, and the SoftFoot Pro, respectively. The NCoT parameter exhibited values of 5.4 and 5.6 [J/(kg*m)] for the Triton and the SoftFoot Pro, respectively, approximately $30\%$ lower than the value reported for the original SoftFoot (7.9 [J/(kg*m)]). Furthermore, the RER value remained below one throughout the physical activity for all three prosthetic feet.

**Table 3:** Key metabolic parameters assessment for the second circuit lap. Comparison among the three tested prosthetic feet

Parameters	Prosthetic feet		
	Triton	Original SoftFoot	SoftFoot Pro
VO_2__plateau_ [mL/(kg*min)]	**18**.**8**	19.9	**18**.**7**
RER_plateau_	0.9	0.9	0.9
NCoT [J/(kg*m)]	**5**.**4**	7.9	**5**.**6**
HR_plateau_ [beats/min]	145	142	147

Among the different prosthetic feet, closest values for VO_2_ and NCoT parameters are highlighted in boldfont

### Questionnaires

Mean values and variability of the responses for each of the nine validated PEQ scales are presented in Table 4. The mobility scale, which includes ambulation and transfer tasks, yielded values of 72.1 $\pm$ 11.2, 70.4 $\pm$ 10.4, and 66.4 $\pm$ 10.6 for the SoftFoot Pro, Triton and original SoftFoot, respectively. Identical scores are recorded for all three prosthetic feet on the Frustration (76.9 $\pm$ 7.4), Perceived Response (94.3 $\pm$ 12.7), Residual Limb Health (31.8 $\pm$ 10.3 ), and Social Burden (76.8 $\pm$ 32.8) scales.

The basic, advanced, and total LCI-5 scores are recorded and reported in Table 5. Each prosthetic foot received a basic mobility score of 26 out of 28, an advanced mobility score of 25 out of 28, and a total score of 51 out of 56.

**Fig. 9:**
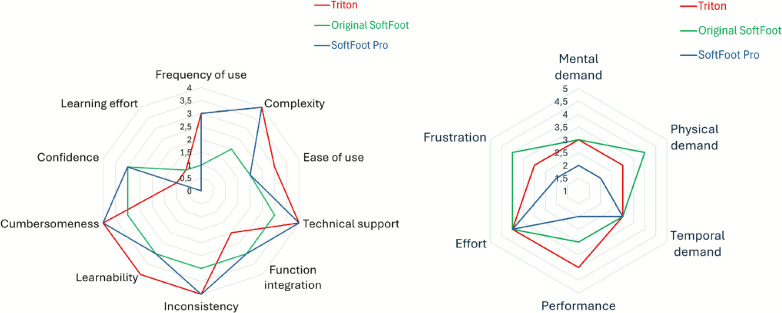
System Usability Scale and NASA Task Load Index results: on the left, SUS questionnaire values for each of the questions; on the right, NASA TLX questionnaire values for each of the six independent questions. Patterns are reported for the Triton (red), the original SoftFoot (green) and the SoftFoot Pro (blue)

SUS results showed usability scores of 75, 60, and 75 out of 100 for the Triton, the original SoftFoot, and the SoftFoot Pro, respectively. For all three prosthetic feet, final values for each question of the questionnaire are reported on the left side of Fig. 9.

**Table 4:** Prosthetic Evaluation Questionnaire results: mean and standard deviation of the responses for the nine validated scales of the PEQ across the three tested prosthetic feet. The evaluation can not be performed for the Sounds scale

Scales	Prosthetic feet		
	Triton	Original SoftFoot	SoftFoot Pro
Mobility (ambulation and transfer)	70.4 $\pm$ 10.4	66.4 $\pm$ 10.6	72.1 $\pm$ 11.2
Appearance	60.1 $\pm$ 27.6	57.9 $\pm$ 25.9	58.3 $\pm$ 26.1
Frustration	76.9 $\pm$ 7.4	76.9 $\pm$ 7.4	76.9 $\pm$ 7.4
Perceived response	94.3 $\pm$ 12.7	94.3 $\pm$ 12.7	94.3 $\pm$ 12.7
Residual limb health	31.8 $\pm$ 10.3	31.8 $\pm$ 10.3	31.8 $\pm$ 10.3
Social burden	76.8 $\pm$ 32.8	76.8 $\pm$ 32.8	76.8 $\pm$ 32.8
Sounds			
Utility	70.3 $\pm$ 13.8	68.8 $\pm$ 13.4	69 $\pm$ 13.3
Well-being	73.2 $\pm$ 2.2	70 $\pm$ 2.3	73.2 $\pm$ 2.2

**Table 5:** Locomotor Capability Index results: basic, advanced and overall LCI-5 scores across the three tested prosthetic feet

	Prosthetic feet		
	Triton	Original SoftFoot	SoftFoot Pro
Basic LCI-5	26	26	26
Advanced LCI-5	25	25	25
Overall LCI-5	51	51	51

Results from the five-point Likert Scale NASA TLX survey for all three prosthetic feet are summarized on the right side of Fig. 9. The SoftFoot Pro is associated with the lowest reported values—2 out of 5—for mental demand, physical demand, performance effort, and frustration experienced by the participant. A peak value of 4 was reported for performance effort when using the Triton, while peak values of 4 were recorded for frustration and physical demand with the original SoftFoot.

### Cybathlon performance

Table 6 reports the completion times recorded by the SoftFoot Pro team and the other three finalists in the final race. Bench and Table tasks’ time is also reported, while it was not in our earlier analysis.

The SoftFoot Pro team achieved second place, following the first-place finish by the Rehab Tech Leg team.^1^ Both teams delivered outstanding performances, each failing only one task: Cross Country for SoftFoot Pro and Balance Beam for Rehab Tech Leg. Notably, they were the only teams in the final to successfully complete the Stairs task.

All teams completed the Slopes, Bench and Table, High Step, and Ladder tasks. In contrast, the Balance Beam, Stairs, and Cross Country tasks were the most challenging, with only two teams managing to complete them.

**Table 6:** Results of the Leg Prosthesis Race final round at Cybathlon 2024. The table shows the performance of Team SoftFoot Pro and of the other three finalist teams

Tasks	Competing teams			
	SoftFoot Pro	Rehab Tech Leg	Ossur LX	Ossur bionics
Balance Beam	43	*X*	**20**	*X*
Stairs	34	**27**	*X*	*X*
Step Over	38	**33**	*X*	44
Slopes	25	22	**21**	32
Bench and Table	**17**	25	21	20
Wobbly Steps	7	**6**	**6**	*X*
High Step	13	**12**	13	16
Ladder	19	18	**16**	26
Cross Country	*X*	**19**	20	*X*
Hurdles	30	**15**	25	*X*

For each task, the shortest completion times are highlighted in boldfont, while italicfont crosses are reported for failed tasks

The SoftFoot Pro pilot delivered an outstanding performance in the Bench and Table task and recorded times comparable to the overall winner in the Wobbly Steps, High Step, and Ladder tasks. However, it was slower in the Stairs, Step Over, Slopes, and Hurdles tasks, ultimately completing the course in 226 s compared to Rehab Tech Leg’s 177 s. The Össur teams failed more tasks: Össur LX failed two tasks (Stairs and Step Over), while Össur Bionics failed five (Balance Beam, Stairs, Wobbly Steps, Cross Country, and Hurdles), placing third and fourth, respectively.

#### User observations

User feedback on the original SoftFoot and SoftFoot Pro was collected during training for Cybathlon 2024 and through post-competition comments. The pilot described the event as “a showcase to the world to present the SoftFoot project” expressing pride in completing the race and aiming to win. He viewed the project as a way to overcome limitations of conventional prosthetic feet and “return to the past” by regaining abilities like walking on uneven terrain and jumping. Regarding the wearability of the three prosthetic feet, the participant reported “No major differences were noted in ease of donning between the SoftFoot devices and the carbon fiber foot as this was influenced more by the socket than the foot design”. The pilot found the Triton — his daily-use prosthesis — better for slippery or unpredictable conditions due to familiarity, and also appreciated its lighter weight. However, he noted that the SoftFoot Pro’s energy return made it feel easier to lift during walking. Regarding the training pre-competition, he said “Initially, adapting to the SoftFoot devices was challenging, but with use, the SoftFoot Pro showed clear advantages in complex tasks, especially those involving agility such as squatting or navigating obstacles”.

In the end, reflecting on the race, the pilot believed that his performance with the SoftFoot Pro had been superior to what he could have achieved with the Triton, based on his experience using the former in the race and the latter in daily life. He also noted its potential usefulness beyond competition, addressing similar challenges encountered in daily life.

Main kinematic, spatio-temporal and metabolic outcomes, for each of the three prosthetic feet, resulting from the completion of the second circuit lap, are summarized in Table 7.

**Table 7:** Summary table reporting the mean values of key metrics for the three prosthetic feet during the second circuit lap (PL = prosthetic limb; IL = intact limb)

	Prosthetic feet		
	Triton	Original SoftFoot	SoftFoot Pro
ANKLE KINEMATICS (PL)			
Level Walking			
mmPlantarflexion peak [°] (early stance)	6	2	**8**
mmDorsiflexion peak [°] (terminal stance)	13	8	**19**
mmRange of Motion [°]	19.9	10.4	**27.8**
Stair ascent			
mmRange of Motion [°]	5.1	7.8	**8.4**
Stair descent			
mmRange of Motion [°]	7	10.4	**15.2**
SPATIO-TEMPORAL PARAMETERS			
Stride Length/BH [%] PL	**68**.**2** $\pm$ **4**.**0**	60.1 $\pm$ 1.6	**71**.**0** $\pm$ **3**.**7**
IL	**69**.**3** $\pm$ **1**.**5**	60.3 $\pm$ 1.2	**70**.**7** $\pm$ **2**.**7**
Gait Speed/BH [% $s^{-1}$]	**54**.**5** $\pm$ **3**.**7**	43.2 $\pm$ 2.6	**54**.**3** $\pm$ **5**.**4**
Total completion times [s]	310	295	**290**
METABOLIC COST			
VO_2__plateau_ [mL/(kg*min)]	**18**.**8**	19.9	**18**.**7**
RER_plateau_	0.9	0.9	0.9
NCoT [J/(kg*m)]	**5**.**4**	7.9	**5**.**6**

Values corresponding to the prosthetic foot exhibiting the greatest plantarflexion and dorsiflexion peak ankle angle, as well as the largest range of motion (ROM), are highlighted in boldfont. The closest values for the spatio-temporal and metabolic parameters are also highlighted, as in Tables 1 and 3, respectively

## Discussion

The 2024 edition of the Cybathlon acted as a catalyst for advancing research, pushing the mechanical innovations of the original SoftFoot, and ultimately leading to the development of the latest state-of-the-art model of the SoftFoot Pro. Moreover, the event provided a unique opportunity to evaluate the SoftFoot Pro in a competitive environment, alongside both research and commercially available prosthetic foot systems. Both the qualification phase and the final round of the competition provided a tangible opportunity to showcase the innovation of the SoftFoot Pro in terms of adaptability to various obstacles. The prosthesis also proved highly versatile, enabling the pilot to negotiate the full range of tasks and challenges presented by the race effectively. Moreover, the prosthesis worked seamlessly with a commercial knee, with no need for any specific adjustment. In qualifications, the pilot scored the maximum 100 points, completing all tasks successfully and placing second only due to a slower time. In the final, the pilot again delivered an excellent performance, earning 90 points, the same as the winning team, and securing second place. This result reflected the mechanical improvements of the SoftFoot Pro, the team’s efforts during training, and the pilot’s commitment, consistency, and perseverance.

In preparation for the competition, the participant, an individual with a unilateral transfemoral amputation, underwent extensive training with the SoftFoot Pro, the original SoftFoot, and a commercial carbon fiber foot. The extensive familiarization period with both SoftFoot models ensured a fair and informed comparison between prosthetic solutions. The participant reported clear advantages with the SoftFoot Pro over both the original SoftFoot and his habitual commercial prosthesis, and chose it as his preferred device for the official competition.

### Level walking

The kinematic analysis of level walking was performed during a training session by assessing the ankle, knee, and hip angles in the sagittal plane. The subject walked using the three prosthetic feet: his commercial carbon fiber foot (a Triton by Ottobock), the original SoftFoot, and the SoftFoot Pro. Each prosthesis resulted in distinct movement patterns. However, all devices shared a common limitation, which consisted of the reduction or absence of plantarflexion during the push-off phase. Such a limitation compromises forward propulsion and overall gait efficiency. Additionally, all devices consistently exhibited a lack of dorsiflexion during the swing phase, resulting in reduced foot-ground clearance compared to the biological data. This was attributed to the fact that all the tested devices were passive and lacked an ankle mechanism capable of generating active torque. The subject tends to increase hip and knee flexion as a compensatory strategy, altering the biomechanics of walking. This is evident in the excessive hip flexion (see Fig. 4) compared to unimpaired subjects, further confirming an inefficient and asymmetric gait.

Although we highlighted these limitations across all tested prosthetic devices, some important differences emerged among them, potentially resulting in different performance levels for each foot. One key distinction of the SoftFoot Pro lies in the insertion of an ankle joint, which is connected to the pylon via a shank link. This configuration allows the rear and anterior arches to rotate more freely around the ankle joint, enabling a greater range of motion relative to the prosthetic pylon. As a result, this increased mobility facilitates the generation of greater torque in response to the force applied by the user during walking. Additionally, the SoftFoot Pro enables a more efficient reverse transmission of the mechanical action from the springs back to the user. Evidence supporting this can be seen in Fig. 4, which shows increased plantarflexion and dorsiflexion at the beginning ($6\%$) and near the end of the stance phase ($45\%$), compared to the other two prosthetic feet. This enhanced motion allows for a more natural and symmetric gait, more closely replicating the plantarflexion and dorsiflexion peaks during stance observed in the sound ankle and resembling the pattern of an unimpaired subject. In contrast, the absence of an ankle joint in the original SoftFoot limits the independent rotation of the arches relative to the pylon, resulting in a restricted range of motion (reduced plantarflexion and dorsiflexion peaks) throughout the stance phase. Consequently, the gait produced with this foot appears less symmetric, with differences in amplitude, particularly during the dorsiflexion phase at the end of the stance, showing $8^{\circ }$ in the prosthetic ankle compared to $13^{\circ }$ in the sound ankle.

Another challenge faced by the prosthetic user, with the previously described prosthesis and the three prosthetic feet tested, is the absence of an early-stance knee flexion peak in the prosthetic limb, which is essential for shock absorption in unimpaired gait and may indicate reduced dynamic balance during walking (see Fig. 4). Furthermore, the slight but unexpected knee hyperextension observed just before the swing phase (approximately 30%–50% of the gait cycle) when the user is wearing the original SoftFoot, may represent a compensatory strategy adopted by the user to counteract the limited dorsiflexion amplitude achieved by the corresponding ankle joint during the same phase of stance. However, further studies are needed to confirm and more thoroughly characterize this compensatory mechanism.

In light of the considerations outlined above, we can observe that, when the user is wearing the original SoftFoot, greater differences between the sound and prosthetic limbs emerge in both the amplitude and pattern of joint angles — particularly at the more distal joints.

This increased asymmetry in limb behavior results in reduced overall gait efficiency, a less symmetric gait pattern, and higher energy expenditure. These findings are supported by the metabolic analysis, which shows higher oxygen consumption (VO_2_), and Net Cost of Transport (NCoT) values associated with the use of the original SoftFoot. This is an expected result, as the original SoftFoot is missing an energy-storing and returning cycle, thus providing a very poor torque during the second half of stance, compared to the Triton, which acts as a spring, and the SoftFoot Pro, which exploits the agonist–antagonist mechanism.

Furthermore, the limited range of plantarflexion and dorsiflexion during stance using the original SoftFoot explains the small ROM value experienced at the prosthetic ankle, compared to the Triton foot and the SoftFoot Pro. Additionally, the lack of energy storage and return in the original SoftFoot determines a lack of propulsive contribution during push-off, contributing to the shorter stride length and walking velocity, as reported in Table 1. In contrast, the aforementioned freedom of relative motion between the rear and front arches and the pylon, enabled by the ankle joint of the SoftFoot Pro, provides greater ankle mobility during gait not only compared to the original SoftFoot, but also surpassing that of the Triton, as shown in Fig. 5. When adopting the SoftFoot Pro, the subject demonstrates stride length and average gait speed comparable with those of the Triton and greater than those achieved with the original SoftFoot. These effects are attributed to the springs of the agonist–antagonist ankle. Together, these components allow for greater energy storage during the stance phase and a more efficient return during push-off compared to the original SoftFoot, enhancing forward propulsion.

### Stairs task

The stairs task was the only Cybathlon task with an in-depth kinematic analysis of the lower limb joint, as it is considered one of the standard gait modes in the literature. When analyzing the findings for the unimpaired subject and the performance of the carbon fiber Triton foot, the kinematic results for both stair ascent and descent were consistent with findings previously reported in the literature [40, 41]. Regarding the sagittal joint angle patterns of the original SoftFoot and SoftFoot Pro, the observed trends align with those documented in the literature, while still preserving the benefits associated with the soft sole design. According to user feedback, during stair descent, the SoftFoot designs offer enhanced control, thanks to the flexible toes that adapt to the geometry of the steps. In stair ascent, although the Triton foot initially required less effort due to its higher energy return compared to the original SoftFoot, this advantage was offset by the performance of the SoftFoot Pro. Thanks to its greater ankle range of motion, the SoftFoot Pro ultimately emerged as a potentially more suitable option for stair ascent. As a general observation, the tested prostheses have a marginal impact on proximal joint behavior. As we can deduce from Fig. 4, both during the ascent and the descent phases, the three prosthetic feet exhibited sagittal plane joint angle patterns at the prosthetic limb with increased differences as the joints are more distal. Kinematic results highlighted that the most pronounced differences among the three prosthetic feet were observed at the prosthetic ankle during the stance phase of the descent task, particularly between 0% and 40% of the gait cycle, where dorsiflexion varied notably in both angle trend and peak values across steps and between devices. However, the data variability among the three steps for each foot, mainly observable at the prosthetic knee and hip during stairs ascent, affects all joint angle patterns. It results in significant changes and discrepancies in the gait cycle events timing. The explanation of this variability can be the subject’s approach to the task, aiming to complete it as quickly as possible while avoiding dropping the cup or committing any other infractions that could lead to disqualification, rather than focusing on the precise repetition of the movement.

Furthermore, the increased range of motion observed at the joints of the sound limb — particularly at the intact ankle — during stair ascent and descent suggests a compensatory strategy to counteract the limited mobility of the prosthetic ankle across all three feet. This finding is consistent with typical hip compensation strategies in individuals with transfemoral amputation, where the absence of a propulsive push-off phase, due to reduced plantarflexion and lack of active knee, is offset by a greater load on the sound limb and increased hip flexion on the prosthetic side to facilitate proper foot placement on the next step. In the Cybathlon setting, this compensation is amplified by the pilot’s focus on speed of task completion over optimal use of prosthetic ROM, contributing to high variability in joint angles across steps and devices. Despite reduced ankle ROM in all prostheses, the SoftFoot Pro still showed the highest ROM during both ascent and descent.

In general, the analyses of the transitions from walk to stair ascent and from stair descent to walk revealed joint-angle trends similar to those observed during the central-step ascent and descent. However, because the task required the participant to place and pick up a tray with a cup from the table before ascending the staircase again, the first step of stair climbing was initiated from a stationary position. This likely explains the reduced knee and hip flexion observed in both the prosthetic user and the unimpaired subject, compared to the central-step ascent. In the transition from stair descent to walk, the absence of plantarflexion at the left ankle of the unimpaired subject at the end of the swing phase can be attributed to the foot being oriented not in the normal forward-walking direction but in preparation for approaching the table. This altered the typical sagittal-plane plantarflexion pattern. Another notable effect was the pronounced plantarflexion observed between 10% and 40% of the gait cycle in two of the three steps performed with the SoftFoot Pro. This may be related to the way the prosthetic user negotiated the stairs: he placed the heel at the step edge, letting the forefoot drop, and moving past the step by shifting the body weight forward, to complete the task more rapidly.

### Cybathlon

The kinematic and metabolic evaluations conducted on the Cybathlon tasks from the 2024 Cybathlon edition focus on assessing performance in 9 out of the 10 tasks with the three prosthetic feet. The fact that the subject performs the circuit at race speed in preparation for the Cybathlon event, adhering to the competition rules, reduced, indeed, the repeatability of the tasks, making a task-specific kinematic analysis complex. Moreover, the scope of this study was to provide an overview of the prosthetic behavior during daily-life tasks, and not to analyze a specific activity.

### Maximum angular excursion

Angular excursion analysis across Cybathlon tasks shows that, in most cases, the SoftFoot Pro consistently outperforms the original SoftFoot and the Triton at the prosthetic ankle. However, differences are less evident in the High Step and Cross Country tasks, which prioritize stability over ankle motion. In the High Step task, the foot starts flat on the ground and finishes flat on the obstacle when stepping on it, while in the Cross Country task, precision of movement is required to avoid touching the floor, a condition that would invalidate the task. For this reason, the use of ankle ROM was limited, and the differences between devices were minimized. The elevated peak hip flexion across Cybathlon tasks, compared to typical level walking, is due to trunk bending for object pickup and high thigh elevation to clear obstacles. This is supported by the noticeably lower hip flexion in the Wobbly Steps task, which involves only walking on unstable surfaces without object handling or obstacle clearance.

### Completion times

The SoftFoot Pro enabled faster walking than the original SoftFoot Pro and matched the Triton in level walking speed. However, its adaptability, combined with the energy recycling, led to faster overall performance in diverse daily activities, as shown by shorter Cybathlon track completion times — even when compared to the Triton. It outperformed the other feet in nearly all individual tasks, except for the Stairs, Ladder, and Cross Country tasks, where the strategy adopted to complete the task and the precision in lower limb movements mattered more than speed.

### Metabolic cost

Finally, the SoftFoot Pro was analyzed in terms of the energy cost required by the subject to complete the experiment, considering both the resting phase and the execution of two laps of the Cybathlon circuit. The presence of springs that mimic the function of human muscles and tendons allows for energy storage during the initial part of the stance phase, replicating the action of dorsiflexor muscles—primarily the tibialis anterior—and enables increased energy release during push-off, emulating the function of plantarflexor muscles and the Achilles tendon. The use of the SoftFoot Pro resulted in an energy expenditure for the user that matched that required when using the Triton, as indicated by similar values of oxygen consumption (VO_2_) and net metabolic cost of transport (NCoT), and lower than the energy expenditure observed with the original SoftFoot. Its design feature, together with the observed results, supports the hypothesis that the energy-recycling mechanism of the agonist–antagonist joint reduces user effort and the need for compensatory movements compared to the original SoftFoot. Moreover, all three prosthetic feet showed a RER value consistently lower than 1, indicating that the subject produces less carbon dioxide than the oxygen consumed to complete the experiment, suggesting a predominant reliance on aerobic metabolism.

### Subjective feedback

The results of this study are consistent and further supported by the participant’s informal feedback, as well as by the outcomes of the evaluation questionnaires. Among the administered questionnaires, the LCI-5 and PEQ include items specifically related to locomotor abilities. Notably, the most promising outcomes emerge from the PEQ, where the user reported greater mobility impressions when using the SoftFoot Pro. Additional insights are provided by the NASA-TLX questionnaire, where the user indicated lower mental and physical demand when walking with the SoftFoot Pro compared to the other devices.

### Study limitations and future directions

In this study, we tested only one subject, who also served as the pilot for our team at Cybathlon. The single-subject design, together with the limited number of repetitions per task, reduces the generalizability and reliability of the conclusions. For this reason, the nature of the study should be regarded primarily as exploratory rather than comparative - an observational analysis of the performance of the three prosthetic feet. To strengthen the findings of this pilot investigation, future studies should include a larger number of subjects, enabling statistical analysis to robustly support the conclusions.

Furthermore, all three prosthetic feet were tested on the same day. This may represent a limitation of the present work, as the fatigue accumulated by the prosthetic user during the testing session might have influenced the metabolic analysis results. In future studies, the metabolic performance of each prosthesis will be assessed on separate days. Additionally, to address the limitations of this study related to data variability and the reduced repeatability of the stair ascent and descent task, future evaluations should consider the use of a staircase with a greater number of consecutive steps. Future developments of this research will include the integration of electromyographic (EMG) analysis to assess lower limb muscle activation in relation to the metabolic cost of performing Cybathlon tasks.

Despite the SoftFoot Pro being designed according to the biomechanical requirements of level walking, it showed a consistent performance across the diverse activities of the Cybathlon 2024 Leg Race. This might stem from the combination of the adaptability to the ground, the implementation of an ankle joint, and the agonist–antagonist mechanism. Future work will also include a dynamic analysis of prosthetic users adopting the SoftFoot Pro to quantitatively evaluate torque and power profiles obtained through the agonist–antagonist mechanism.

## Conclusion

In this paper, we analyzed and compared the kinematics, metabolic cost, and performance of a single subject performing the Cybathlon 2024 Leg Race, with three different prosthetic devices: the Triton foot, the original SoftFoot, and the SoftFoot Pro. The agonist–antagonist SoftFoot Pro demonstrated significant advancements over the original SoftFoot, combining enhanced mechanical functionality with improved metabolic efficiency. This device was designed to add the energy recycling property of the carbon fiber feet, keeping the adaptability of the original SoftFoot. Its use during both the completion of challenging tasks from the Cybathlon 2024 competition and level walking highlighted an increased ankle joint range of motion enabled by the integrated revolute joint. Furthermore, the energy storage and return feature, enabled through anterior and posterior springs mimicking the function of agonist–antagonist muscles and tendons, contributed to a noticeable reduction in the subject’s effort, improving overall metabolic performance. This was supported by lower oxygen consumption (VO_2_) and net metabolic cost of transport (NCoT), which were comparable to the values obtained with the subject’s regular carbon fiber foot and significantly better than those recorded with the original SoftFoot. The participation of the SoftFoot Pro in the official Cybathlon 2024 Leg Prosthesis Race, along with the excellent performance of its pilot, further highlighted the potential of this experimental device. These results, supported by the user’s observations and qualitative feedback, suggested that the SoftFoot Pro could represent a promising alternative to existing commercial energy-storing and returning carbon fiber prosthetic feet, combining the energy recycling property with the adaptability to the ground.

## Supplementary information

Supplementary file 1. This image illustrates the kinematics of all lower limb joints for both the prosthetic and intact limbs during walk to stair ascent and stair descent to walk transitions (three steps for each prosthetic foot).

Supplementary file 1. This video illustrates the fitting process of the SoftFoot Pro prosthesis, its integration into the participant’s daily life, and its application during both training and the official Cybathlon Leg race competition.

## Data Availability

The dataset generated and the analysis code are available from the corresponding author on reasonable request.

## Abbreviations

SACH: Solid ankle cushioned heel
ESR: Energy storage and return
IMU: Inertial measurement units
PEQ: Prosthetic evaluation questionnaire
LCI: Locomotor capabilities index
SUS: System usability scale
NASA TLX: NASA task load index
LW: Level walking
U: Stair ascent
D: Stair descent
IL: Intact limb
PL: Prosthetic limb
ROM: Range of motion
BH: Body height
RER: Respiratory exchange ratio
NCoT: Net cost of transport
EEq: Energetic equivalent
RMR: Resting metabolic rate
WS: Walking speed
HR: Heart rate
EMG: Electromyography

## Footnotes

1 https://cybathlon.com/en/teams/rehab-tech-leg
