# Supplementary figures and images for: The softfoot pro at the cybathlon: kinematic, metabolic, and user performance evaluation
Source: J Neuroeng Rehabil. 2026 Jan 30;23:78. doi: 10.1186/s12984-025-01862-y (PMC12931035; doi:10.1186/s12984-025-01862-y)

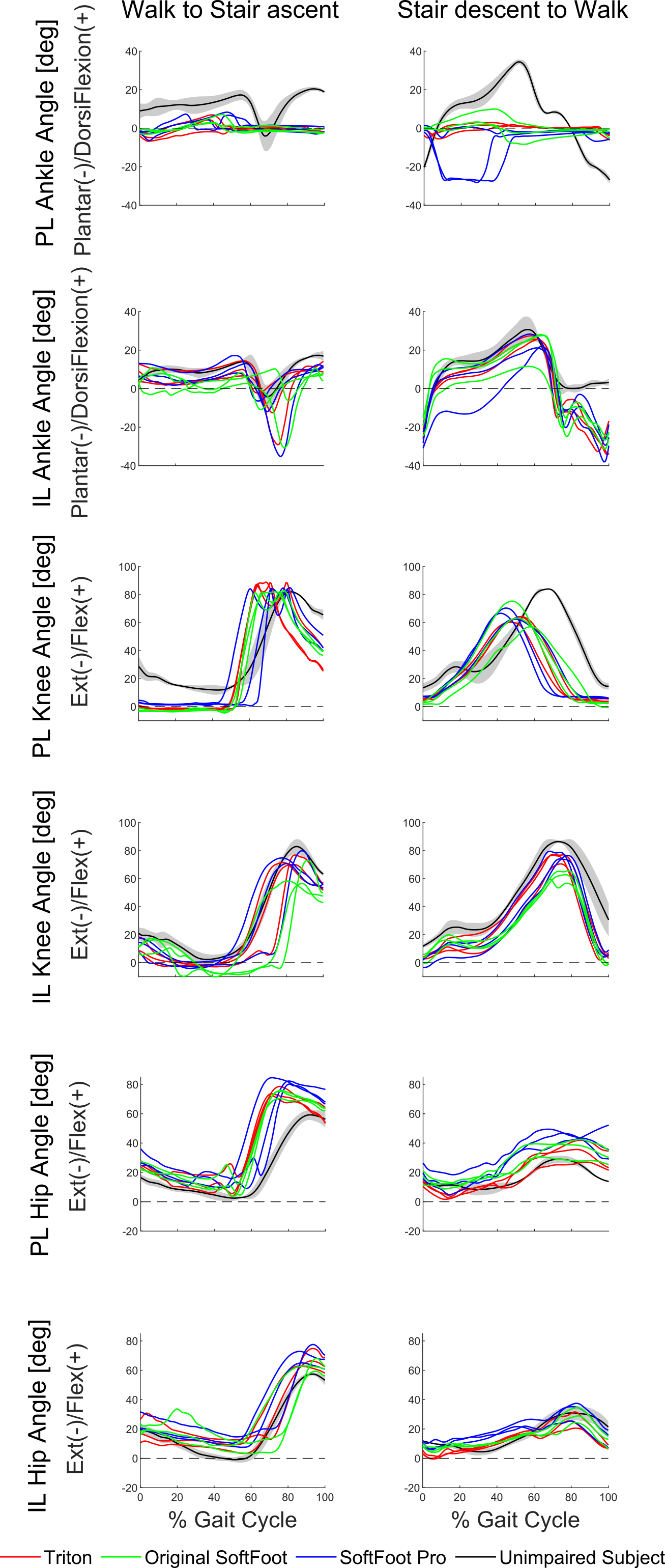

Supplement: Supplementary file 1 — This image illustrates the kinematics of all lower limb joints for both the prosthetic and intact limbs during walk to stair ascent and stair descent to walk transitions (three steps for each prosthetic foot). [file 12984_2025_1862_MOESM1_ESM.pdf]
